# Genomic Insights and Comparative Analysis of Novel *Rhodopseudomonas* Species: A Purple Non-Sulfur Bacterium Isolated from Latex Rubber Sheet Wastewater

**DOI:** 10.3390/life15050754

**Published:** 2025-05-08

**Authors:** Chollachai Klaysubun, Nattarika Chaichana, Sirikan Suwannasin, Kamonnut Singkhamanan, Thunchanok Yaikhan, Duangporn Kantachote, Rattanaruji Pomwised, Monwadee Wonglapsuwan, Komwit Surachat

**Affiliations:** 1 Department of Biomedical Sciences and Biomedical Engineering, Faculty of Medicine, Prince of Songkla University, Songkhla 90110, Thailand; chollachai951@gmail.com (C.K.); naampueng.np@gmail.com (N.C.); sirikan4036@gmail.com (S.S.); skamonnu@medicine.psu.ac.th (K.S.); p_rair@hotmail.com (T.Y.); 2 Division of Biological Science, Faculty of Science, Prince of Songkla University, Songkhla 90110, Thailand; duangporn.k@psu.ac.th (D.K.); rattanaruji.p@psu.ac.th (R.P.); monwadee.wo@psu.ac.th (M.W.); 3 Translational Medicine Research Center, Faculty of Medicine, Prince of Songkla University, Songkhla 90110, Thailand

**Keywords:** purple non-sulfur bacteria, *Rhodopseudomonas*, wastewater treatment, genome, bioinformatics

## Abstract

*Rhodopseudomonas* is recognized for its versatile metabolic capabilities that enable it to effectively degrade pollutants and survive various environmental stresses. In this study, we conducted a genome analysis of *Rhodopseudomonas* sp. P1 to investigate its genetic potential for wastewater treatment processes. Phylogenetic and genome-relatedness analyses confirmed that strain P1 is genetically distinct from other species within the *Rhodopseudomonas* genus, establishing it as a novel species. The genome sequences obtained and analyzed focused on genes related to carbon and nutrient removal, photosynthetic capabilities, nitrate and nitrite reduction, and the biodegradation of common wastewater pollutants. The identification of wastewater treatment-related genes followed an extensive review of the existing literature that helped in selecting genes involved in various wastewater treatment mechanisms. The genome of *Rhodopseudomonas* sp. P1 contains a diverse array of genes involved in carbon and nutrient cycling, pollutant biodegradation, and metal resistance, all of which are crucial for its survival in the complex wastewater environment. Specifically, the strain contains genes responsible for the denitrification, nitrogen fixation, sulfur cycling, and detoxification of toxic metals such as copper and arsenic. These findings highlight the potential application of *Rhodopseudomonas* sp. P1 in wastewater treatment, particularly in environments contaminated with organic pollutants and heavy metals. However, while the genomic features indicate significant promise, the practical implementation of *Rhodopseudomonas* sp. P1 in real-world wastewater treatment systems will require further investigation, optimization, and validation to fully harness its potential for sustainable and efficient wastewater treatment.

## 1. Introduction

*Rhodopseudomonas* is a genus of Gram-negative, purple non-sulfur photosynthetic bacteria belonging to the family Nitrobacteraceae, and it is found in diverse environments [1]. Currently, 10 species within the genus *Rhodopseudomonas* have validly published names (https://lpsn.dsmz.de/genus/rhodopseudomonas, accessed on 17 March 2025). *Rhodopseudomonas* can grow with or without oxygen, fix nitrogen, and degrade various organic compounds such as 2-chlorophenol, 3-methylindole, hexabromocyclododecane, 3-chlorobenzoate, syringic acid, and vanillyl alcohol [2,3,4,5,6]. They exhibit versatile metabolisms, utilizing carbon dioxide or organic compounds as carbon sources and light, inorganic, or organic compounds as energy sources [7], demonstrating their potential utility in bioremediation and environmental management applications.

The use of *Rhodopseudomonas* for wastewater treatment is gaining attention due to its environmental and economic benefits. This genus is commonly applied in both aquaculture and wastewater treatment processes. During treatment, *Rhodopseudomonas* proliferates quickly as it breaks down pollutants while producing valuable by-products such as bacteriochlorophyll, carotenoids, and coenzyme Q10 [8]. Specifically, *Rhodopseudomonas palustris* is capable of utilizing a variety of aromatic compounds for its growth including phenolic, dihydroxylated, and methoxylated aromatic acids, as well as aromatic aldehydes and hydroaromatic acids [9]. Since plants commonly use lagoons or oxidation ponds for wastewater treatment, a major problem with this system is the production of hydrogen sulfide (H_2_S). A study by Kornochalert et al. (2014) [10] found that treating latex rubber sheet wastewater using the *Rhodopseudomonas palustris* strain P1 enhanced by fermented pineapple extract (FPE) could remove chemical oxygen demand (COD), suspended solids, and total sulfide without producing harmful hydrogen sulfide gas. This process also yielded a single-cell protein (SCP) which could serve as animal feed. Furthermore, an analysis of the genome sequence of *Rhodopseudomonas palustris* strain CGA009 indicated a high degree of metabolic flexibility, explaining its capacity to inhabit heterogeneous environments [11]. A genetic analysis of 75 *Rhodopseudomonas* isolates from sediment samples revealed substantial divergences between strains, suggesting that the *Rhodopseudomonas* genus comprises discrete populations each with unique physiological attributes [11]. 

This research focuses on exploring the genomic characteristics of *Rhodopseudomonas* sp. P1, a purple non-sulfur bacterium isolated from wastewater derived from latex rubber sheets, and conducting a comparative analysis on this strain. Phylogenetic and genome-relatedness analyses confirm that this bacterium represents a novel species. It belongs to a group known for its ability to utilize a variety of compounds and adapt to diverse environments. The main goal of this study is to provide a deeper understanding of the genomic basis behind the versatility of this bacterium and its ability to thrive in a unique wastewater habitat.

## 2. Materials and Methods

### 2.1. Preparation of Bacterial Strains and Isolation of Genomic DNA

In a previous study, the isolate P1 was obtained from a wastewater sample collected at a rubber sheet manufacturing plant in southern Thailand [12]. The sample was inoculated into an equal volume of double-strength G-5 broth for isolating purple non-sulfur bacteria (PNSB) and incubated at 28–32 °C under anaerobic light conditions. A single colony of the strain was then purified on G-5 agar and incubated under the same conditions. To extract the genomic DNA, the Presto^TM^ mini gDNA bacteria kit (Geneaid Biotech, Taipei, Taiwan) was utilized following the instructions provided by the manufacturer. The quality of the obtained genomic DNA was evaluated using the NanoDropTM 2000c spectrophotometer (Thermo Fisher Scientific, Norristown, PA, USA).

### 2.2. Whole-Genome Sequencing

For this investigation, the PacBio RSII sequencer provided by Macrogen Inc., Seoul, Republic of Korea, was employed to decipher the complete DNA sequence of the strain P1. Each bacterial strain was sequenced using a single-molecule real-time (SMRT) cell. The resulting sequences amounted to approximately 1.1 Gb, with an average read length of 6.73 kb.

### 2.3. Sequence Analysis and Visualization

To reduce errors from the sequencer, raw sequence reads were corrected using the Canu assembler [13]. These corrected reads were then used for de novo genome assembly via the Unicycler pipeline [14], employing default settings. The completeness of the assembled genomes was evaluated with Busco [15,16]. Genome annotation was performed using the NCBI Prokaryotic Genome Annotation Pipeline (PGAP) [17] and the Bacterial Bioinformatics Resource Center (BV-BRC) [18], offering a comprehensive genetic analysis.

Functional annotations were obtained through the Rapid Annotation using Subsystem Technology (RAST) server [19] and EggNOG 5.0 [20]. Plasmids were identified using the PlasmidFinder database [21], and genomic islands were predicted via the IslandViewer server [22]. The bacterial pathogenicity toward human hosts was assessed using PathogenFinder [23]. Prophages were identified using the PHASTER web server [24]. Biosynthetic gene clusters were analyzed with antiSMASH 7.0 [25] using the “strict” parameter. Carbohydrate-active enzyme gene clusters were identified with dbCAN2 HMMs of CAZy families v10 on the KBase web interface [26,27] with default settings. Finally, the circular genome map was generated using the CGView server [28].

### 2.4. 16S rRNA Phylogeny and Multilocus Sequence Analysis

To perform preliminary species identification, the 16S rRNA gene sequences extracted from the RAST genome annotation were analyzed using the EzBioCloud database [29]. The 16S rRNA gene phylogeny was constructed using 16S rRNA gene sequences from ten *Rhodopseudomonas* type strains and *Cereibacter changlensis* JA139^T^, sourced from the NCBI database. Additionally, the autoMLST (Automated Multi-Locus Species Tree) system was applied using the genomes of seven available *Rhodopseudomonas* type strains. The phylogenetic analysis utilized core genes derived from the genomes with the default settings. *Cereibacter changlensis* DSM 18774^T^ served as the outgroup [30]. The maximum likelihood (ML) tree was generated using the Kimura two-parameter model with 1000 bootstrap replicates. Furthermore, phylogenetic trees based on the neighbor-joining and maximum parsimony methods were also created with 1000 bootstrap replicates. Sequence alignment for all analyses was carried out using MEGA X (v10.2.6) software [31,32].

### 2.5. Comparative Genome Analysis

Species delineation was validated by calculating the overall genome relatedness index, which was based on digital DNA–DNA hybridization (dDDH) values, average nucleotide identity (ANI), and average amino acid identity (AAI). Genomes for strain P1 and the type strains of *Rhodopseudomonas* were examined using the Genome BLAST Distance Phylogeny (GBDP) *d4* formula via the Type Strain Genome Server (TYGS) [33]. All dDDH values were computed using the Genome-to-Genome Distance Calculator (GGDC 3.0) and visualized in a heatmap generated with the Chiplot online tool [34]. The dDDH threshold for species delineation was set below 70%, with values under 79% indicating subspecies delineation [35]. ANI and AAI analyses were conducted using the online tool developed by Kostas Lab [36]. The species delineation threshold was established at less than 95–96% similarity for ANI and AAI, respectively [37,38,39].

For the genome comparison analysis, the Orthovenn3 web server [40] was used to compare strain P1 with the genomes of the closest *Rhodopseudomonas* type strains. Protein sequences for these strains obtained through RAST annotation were uploaded and analyzed using the OrthoFinder algorithm [41] with an inflation value of 2 and an E-value threshold of 1 × 10^−5^.

### 2.6. Wastewater Treatment-Related Genes

The identification of wastewater treatment-related genes in *Rhodopseudomonas* strains was carried out through a comprehensive review of the existing literature. The information gathered from these reviews was then used in our comparative analysis to guide the selection of potential genes associated with wastewater treatment from various mechanisms. Among the gene clusters investigated, those related to wastewater treatment were primarily involved in biodegradation, pollutant removal, nitrogen and sulfur cycling, and metal resistance. Additionally, the KBase tool “Search with HMMs of MicroTrait bioelement families” [42] was used to assess gene families associated with environmental bio-element cycling in strain P1 compared with closely related strains. A heatmap visualizing the results was generated using the ChiPlot online tool (https://www.chiplot.online/, accessed on 17 March 2025).

## 3. Results and Discussion

### 3.1. Genome Profiles of Novel Species

**Figure 1:**
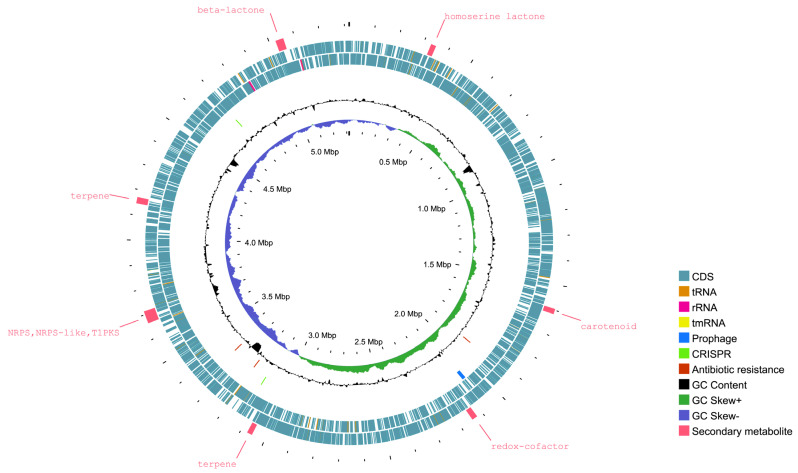
A graphical representation of the circular genome of *Rhodopseudomonas* sp. P1. The outermost circle illustrates the locations of secondary metabolic gene clusters. Moving inward, the map shows the distribution of coding sequences (CDS), tRNA, rRNA, tmRNA, the prophage region, CRISPR-Cas genes, antibiotic resistance genes, and the shift in GC content.

The general features of strain P1 are depicted in Figure 1. The complete genome of this strain consists of a single circular chromosome, with no plasmids identified. The chromosome size is 5.3 Mbp with a GC content of 65.1%. The genome demonstrates a completeness of 99.8% and a contamination level of less than 0.1%, which signifies the exceptional quality and accuracy of the assemblies.

**Figure 2:**
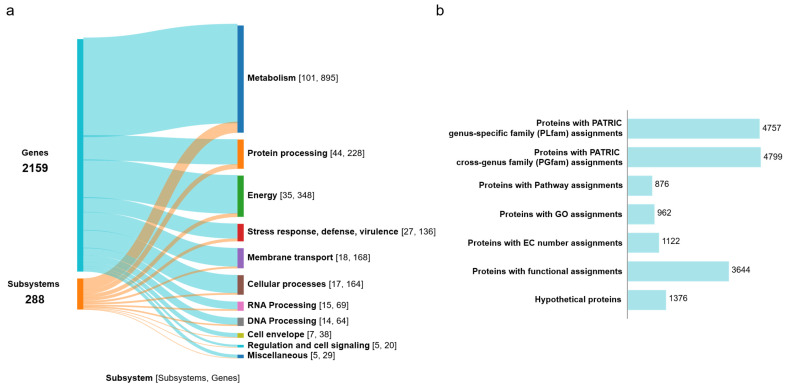
Functional characterization of the P1 genome. (**a**) Sankey diagram illustrating the distribution of genes into subsystems classified by biological functions. (**b**) Summary of protein annotations as provided by BV-BRC.

The functional annotation of the genome illustrated in the Sankey diagram (Figure 2a, Table S1) shows the distribution of 2159 genes across 288 subsystems. Key areas of focus include metabolic functions (895 genes in 101 subsystems), energy production (348 genes in 35 subsystems), protein processing (228 genes in 44 subsystems), DNA processing (64 genes in 14 subsystems), and stress response and virulence (136 genes in 27 subsystems). The annotation identified 1376 hypothetical proteins and 3644 proteins with specific functional roles (Figure 2b). Among the functional proteins, 1122 were associated with Enzyme Commission (EC) numbers [43], 845 with Gene Ontology (GO) assignments [44], and 876 were mapped to KEGG pathways [45]. Moreover, the PATRIC annotation identified 4757 proteins belonging to PLFams and 4799 proteins were associated with PGFams [46]. RAST analysis classified the genes into different subsystem categories based on their predicted functions (Figure S1). The top three subsystem categories in P1 are amino acid derivatives (330 genes), followed by carbohydrates (232 genes) and cofactors, vitamins, prosthetic groups, and pigments (211 genes). Other categories include protein metabolism (187 genes), respiration (157 genes), fatty acid and lipid metabolism (139 genes), nitrogen metabolism (92 genes), membrane transporters (81 genes), and stress responses (76 genes). These subsystems highlight the broad metabolic capabilities of this strain, suggesting its metabolic versatility in adapting to wastewater environments.

**Figure 3:**
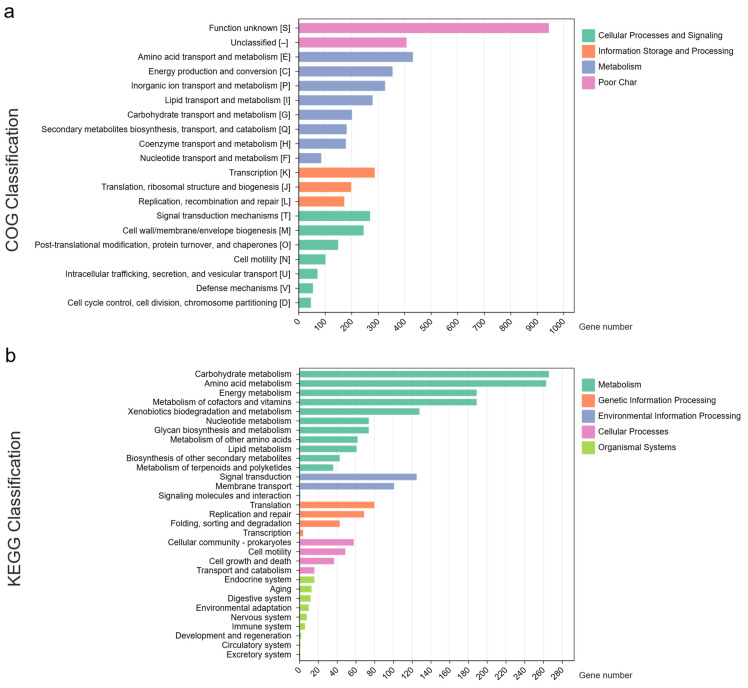
Functional classification of genes predicted by COG and KEGG analysis in the P1 genome. (**a**) COG and (**b**) KEGG classification.

COG analysis revealed a significant proportion of genes associated with amino acid transport and metabolism (432 genes), energy production and conversion (355 genes), and inorganic ion transport and metabolism (327 genes) (Figure 3a). This indicates that the P1 strain possesses an advanced system for transporting and metabolizing various carbon and nitrogen sources, especially those present in wastewater from latex rubber sheet production. KEGG analysis further identified genes associated with various metabolic pathways (Figure 3b), with the highest number (713 genes) related to different metabolic pathways, followed by carbohydrate metabolism (266 genes), amino acid metabolism (263 genes), energy metabolism (189 genes), metabolism of cofactors and vitamins (189 genes), and xenobiotic biodegradation and metabolism (128 genes).

The analysis of the P1 genomes revealed 23 GI-encoded proteins of both hypothetical and known function (Table S2). The GIs contained ribosomal proteins, transposable elements (IS5, IS110), prophages, and stress-related proteins, among others. No genes associated with pathogens, virulence factors, or antibiotic resistance were detected within the GIs. Further analysis of the resistance genes in the P1 genome through the CARD database showed resistance genes involved in multidrug resistance efflux pumps (Figure 1, Table S3). Gene families like small multidrug resistance (SMR) antibiotic efflux pumps and resistance nodulation cell division (RND) antibiotic efflux pumps were identified. The strain P1 was found to have two copies of the *adeF* gene (strict hits) which confer resistance to tetracycline antibiotics by antibiotic efflux. Furthermore, the presence of prophages in the P1 strain may improve adhesion and enhance environmental adaptability and antibiotic resistance (Table S4). Predicted CRISPR sequences were also found with two CRISPR elements (100 and 64 bp). These elements contained one direct repeat of 23 bp and one spacer, but no associated cas-family genes were detected (Figure 1).

In the CAZyme analysis, 112 CAZyme genes were detected in the P1 genome. Among these, glycoside hydrolases (GHs) were the most prevalent with 83 genes, followed by glycosyltransferases (GTs, 20 genes), auxiliary activities (AAs, 18 genes) and carbohydrate esterases (CE, one gene) (Table S5). This distribution highlights the ability of the strain to degrade carbohydrates, a key trait that likely helps it thrive in diverse and challenging environments. Additionally, the analysis of biosynthetic gene clusters (BGCs) revealed that strain P1 contains clusters for homoserine lactone (hserlactone), terpene, redox-cofactor, type I polyketide synthase (TIPKS), non-ribosomal peptide synthetase (NRPS), NRPS-like, and beta-lactone. These BGCs are matched to the production of carotenoids (100% similarity) and bottromycin A2 (6% similarity), as shown in Figure 1 and Table S6.

Different *Rhodopseudomonas* species produce a variety of carotenoids, with the composition varying depending on the species and growth conditions. Research has identified several strains with carotenoid production capabilities including *R. palustris* ATCC 17001 [47], *R. palustris* CGA009 [48], and *R. faecalis* PA2 [49]. The main types of carotenoids identified in *Rhodopseudomonas* include spheroidene and its derivatives, as well as spirilloxanthin. *Rhodopseudomonas* species, particularly *R. palustris* and *R. faecalis*, are also capable of producing lycopene, a valuable red carotenoid with strong antioxidant properties [48,50]. This confirms their ability to biosynthesize lycopene and highlights their potential for sustainable bioproduction. Additionally, the *crtI* gene, found in the P1 genome, has been identified as critical for carotenoid synthesis. Knockout experiments have shown that inactivation of this gene leads to decreased carotenoid production and reduced tolerance to environmental stress [48].

### 3.2. Phylogenetic Relationship Analysis

The complete 16S rRNA gene sequences of strain P1 were retrieved from the RAST genome annotation. These sequences were then analyzed for 16S-based identification using EzBioCloud. The strain showed high sequence similarities to *Rhodopseudomonas thermotolerans* JA576*T* (99.72%), *Rhodopseudomonas pentothenatexigens* JA575^T^ (99.71%), *Rhodopseudomonas palustris* ATCC 17001^T^ (99.65%), *Rhodopseudomonas faecalis* gc^T^ (98.93%), and *Rhodopseudomonas harwoodiae* JA531^T^ (98.82%), indicating a close relationship with these species.

**Figure 4:**
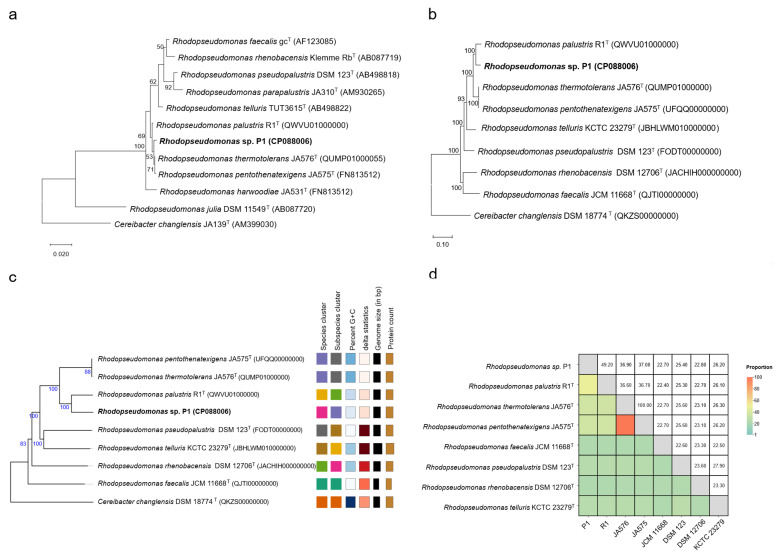
Phylogenetic and genome sequence analysis of *Rhodopseudomonas* strains. (**a**) Maximum likelihood tree based on 16S rRNA gene sequences of strain P1 compared to *Rhodopseudomonas* type strains. *Cereibacter changlensis* JA139^T^ was employed as an outgroup. The bootstrap value (percentage) was computed based on 1000 bootstrap replicates, and values with more than 50% are shown. (**b**) autoMLST genome tree showing the relationship of strain P1 to seven *Rhodopseudomonas* type strains. The tree was visualized using MEGA X, and bootstrap values above 50% are displayed on the tree. (**c**) Genome sequence-based tree from TYGS analysis results for strain P1. *Cereibacter changlensis* DSM 18774^T^ was employed as an outgroup. (**d**) Heatmap with dDDH values between strain P1 and seven *Rhodopseudomonas* type strains.

To further clarify the phylogenetic positions, 16S rRNA gene sequences from ten *Rhodopseudomonas* type strains along with the outgroup *Cereibacter changlensis* JA139^T^ were used to construct phylogenetic trees. As shown in Figure 4a and Figures S2 and S3, strain P1 clustered with *R. thermotolerans* JA576^T^ and *R. pentothenatexigens* JA575^T^ on the same branch, while *R. palustris* R1^T^ formed a distinct branch. Further genome analysis is needed to confirm these findings and clarify the phylogenetic relationships.

### 3.3. Comparative Genome Analysis of Novel Species

Based on the initial findings that strain P1 belonged to the genus *Rhodopseudomonas* from the 16S rRNA gene sequence, whole-genome analysis offers the most precise and comprehensive understanding of evolutionary relationships, providing strong evidence for accurate species classification and identification [51].

The multigene sequence analysis was inferred using the autoMLST pipeline [30]. Ninety housekeeping genes were selected. The gene sequences from strain P1 were aligned with those of seven closely related whole-genome sequences from the database. The analysis revealed that strain P1 formed a monophyletic clade with *R. palustris* R1^T^ (Figure 4b), consistent with phylogenomic analyses based on the TYGS pipeline [33] (Figure 4c). Additionally, the dDDH (49.2%) values between strain P1 and *R. palustris* R1^T^ were below the 70% divergence threshold used for prokaryotic species delineation [52] (Figure 4d). Similarly, ANI (93.2%) and AAI (94.9%) values between the two strains were below the 95% threshold [38,39], further confirming their separation from *R. palustris* R1^T^. These findings suggest that strain P1 represents a novel species within the genus *Rhodopseudomonas*. Furthermore, *R. thermotolerans* JA576^T^ showed 100% dDDH, ANI, and AAI values with strain *R. pentothenatexigens* JA575^T^, indicating that they are the same species.

**Figure 5:**
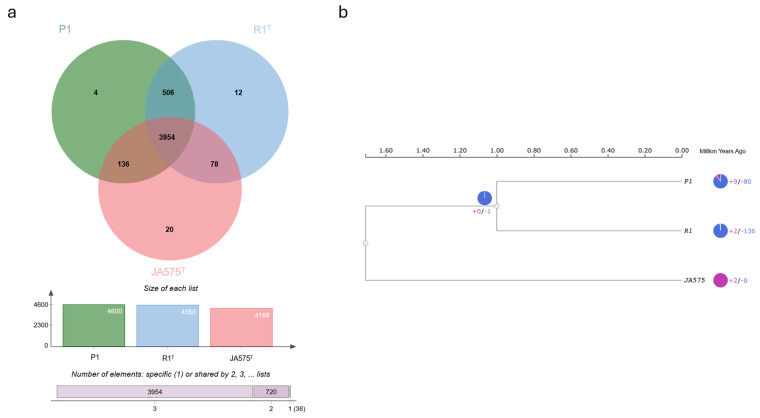
Orthologous cluster identification and comparative analysis. (**a**) Representation of orthologous gene clusters across three selected strains. The Venn diagram shows the unique and shared orthologous gene clusters, with an accompanying bar chart providing a quantitative breakdown of the number of clusters for each strain. (**b**) Analysis of gene family evolution in the three strains, highlighting contracted and expanded gene families. The pie chart visually contrasts the number of contracted (in purple) and expanded (in blue) gene families, illustrating the evolution of gene families and species-specific differences.

The OrthoVenn3 web server was used for whole-genome comparison to examine functional differences among closely related strains. The Venn diagram allowed for a visualization of unique and shared genes or pseudogenes between *Rhodopseudomonas* sp. P1 and related species such as *R. palustris* R1^T^ and *R. pentothenatexigens* JA575^T^. The analysis identified seven overlapping orthologs across 4710 clusters, including 3954 core genome orthologs and 3932 single-copy clusters. In total, 15,182 proteins were identified with 1723 of them classified as singletons, representing 11.35% of the dataset. Singletons were most abundant in *R. pentothenatexigens* JA575^T^, followed by *R. palustris* R1^T^ and *Rhodopseudomonas* sp. P1 (Figure 5a). An ultrametric phylogenetic tree reveals the expansions and contractions of gene families, providing insight into evolutionary trajectories. Compared to their common ancestor, *Rhodopseudomonas* sp. P1 exhibited 9 expanded and 80 contracted gene families, while the closest type of strain *R. palustris* R1^T^ showed expansions in 2 and contractions in 136 gene families. This further supports the genetic divergence between *Rhodopseudomonas* sp. P1 and the closely related type strains (Figure 5b).

### 3.4. Bioelement Cycling and Metal Resistance in Rhodopseudomonas sp. P1

**Figure 6:**
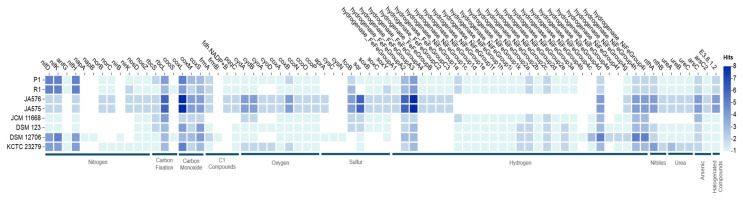
Abundance of MicroTrait bioelement family genes in strain P1 and the type strains of the genus *Rhodopseudomonas*.

The microTrait database [42] was used to map genes into ecological guilds, providing insights into the strain’s potential metabolic flexibility. The analysis of bioelement cycling genes including nitrogen, carbon fixation, carbon monoxide, C1 compounds, oxygen, sulfur, hydrogen, nitriles, arsenic, and halogenated compounds in strain P1 revealed similarities with other strains in the *Rhodopseudomonas* genus with some variations (Figure 6, Table S7). Strain P1 has bioelement cycling genes close to *R. palustris* R1^T^. The genomic analysis of P1 highlights its potential for denitrification (*nifD, nifK*, *nifH, anfG*) [53,54], oxygen utilization (*cydA, cydB, cyoA, cyoD, coxA, coxB, ccoN, ccoO, ccoP*) [55], sulfur cycling (*fccB, sqr, soxB, soxC, soxY*) [56,57], and diverse hydrogen metabolism (FeFe and NiFe hydrogenases) [58,59,60]. This versatility allows the strain to efficiently adapt to different oxygen conditions and resource availability, making it well-suited for wastewater treatment applications.

Wastewater from rubber sheet processing contains ammonia, formic acid, sodium metabisulfite, and sodium sulfide [12]. The plants commonly use lagoons or oxidation ponds for wastewater treatment. The toxic gas H_2_S presents a major problem for the system. However, elemental sulfur (S^0^) is considered the most suitable product for H_2_S conversion due to its hydrophobic nature and relatively low toxicity in the environment [61]. Sulfide–quinone oxidoreductase (SQR) is a flavoprotein enzyme critical for converting toxic H_2_S into S^0^. Similarly, Thi Phuc et al. (2024) [62] demonstrated that the SQR enzyme encoded by the *sqr* gene from *Rhodopsedomonas palustris* PAM 36 exhibits optimal activity at pH 6.5 and 30°C, with sulfide oxidation rates reaching 20–21 U/mL in membrane fractions. The presence of the *sqr* gene in the P1 genome, which is consistent with previous in vitro studies [10], confirms that this strain could be promising for application in the bioremediation of H_2_S pollution.

Additionally, strain P1 contains a higher number of urease genes (*ureA, ureB, ureC*) [63,64], enhancing its ability to use urea as a nitrogen source and increasing nitrogen utilization efficiency in a range of conditions. The presence of nitrile hydratase genes (*nthA, nthB*) [65,66] enables the strain to utilize nitriles as both a carbon and nitrogen source, which is beneficial for survival in environments with industrial waste or contaminated soils. The presence of arsenic reduction genes (*arsC, arsC2*) [67,68] and haloacid dehalogenase (E3.8.1.2) [69,70] highlights the strain’s ability to detoxify halogenated organic pollutants by cleaving the carbon–halogen bond, aiding in the removal of chemical stressors from the environment.

Based on the BV-BRC annotation, P1 contains a variety of metal resistance genes that contribute to its ability to tolerate and remove heavy metals from wastewater. The *czc* operon in the P1 genome includes three structural genes (*czcC*, *czcB*, and *czcA*) forming a metal-transporting resistance nodulation division-type (RND-type) efflux system. This system functions to transport divalent cations and protons, detoxifying Zn^2+^, Co^2+^, and Cd^2+^ (EC 3.6.3.3) [71]. The P1 genome also includes copper-translocating P-type ATPases (CopA) and copper resistance proteins (CopD and CopC), which are responsible for the transport of copper from the cytoplasm and copper detoxification under aerobic conditions [72]. The *cus* operon also aids in copper resistance under anaerobic conditions [73].

Furthermore, the P1 genome contains the *arsRBC* operon, which includes *arsR* (a transcriptional regulator), *arsB* (an integral membrane protein), and *arsC* (an arsenate reductase that converts arsenate to arsenite) [74,75]. Evidence of an arsenite S-adenosylmethionine methyltransferase gene (*arsM*) suggests that the strain uses this pathway for arsenic resistance through methylation and volatilization [76]. Additionally, genes for metal uptake including a P-type ATPase involved in magnesium uptake (EC 3.6.3.2) [77] and a high-affinity nickel transporter gene (*hoxN*) [78] further enhance the strain’s ability to handle metal stress in wastewater treatment.

*Rhodopseudomonas* species demonstrate significant potential for heavy metal removal in wastewater treatment, particularly when combined with complementary microbial systems. Recent research investigating the effect of varying Fe^2+^ concentrations on pollutant removal in heavy oil refinery wastewater treatment using *Rhodopseudomonas* and *Pseudomonas* showed that an optimal Fe^2+^ concentration of 20 mg/L significantly enhanced treatment performance, achieving 73.51% removal of solute COD and 92.26% removal of ammonia. Additionally, this dosage yielded quantities of valuable biomolecules such as carotenoids, bacteriochlorophyll, and coenzyme Q10 [79]. Similarly, the study evaluated the effects of metal ions on *Rhodopseudomonas palustris* growth and 5-aminolevulinic acid (ALA) production during wastewater treatment, demonstrating that specific metal ions such as Mg^2+^, Fe^2+^, Co^2+^, Ni^2+^, and Zn^2+^ enhanced COD removal, biomass growth, and ALA yield [80], further confirming their versatility in wastewater treatment.

## 4. Conclusions

In this study, we conducted a comprehensive genomic analysis of *Rhodopseudomonas* sp. P1, a purple non-sulfur bacterium isolated from wastewater, specifically from latex rubber sheets. The results of this genomic investigation not only provide a deeper understanding of the metabolic potential of this bacterium but also confirm that strain P1 represents a novel species within the genus *Rhodopseudomonas*. Through genome-relatedness analyses and phylogenetic studies, it was established that *Rhodopseudomonas* sp. P1 is genetically distinct from other known species based on ANI, AAI, and dDDH values, all of which were below the thresholds for species delineation. Functional annotation of the genome identified a broad range of genes involved in carbon and nutrient cycling, stress responses, pollutant biodegradation, and metal resistance, which are essential for its survival in the challenging environment of wastewater Notably, *Rhodopseudomonas* sp. P1 demonstrates significant potential for H_2_S bioremediation because of the presence of the sulfide–quinone oxidoreductase gene, which confirms its ability to convert toxic H_2_S into elemental sulfur and is consistent with previous in vitro studies. Additionally, strain P1 has genes associated with the utilization of urea, nitriles, and arsenic compounds. The presence of metal resistance genes highlights the suitability of strain P1 for heavy metal removal from wastewater. Overall, the novel species *Rhodopseudomonas* sp. P1 demonstrates a versatile genetic profile and metabolic capabilities, making it an ideal candidate for sustainable and effective wastewater treatment applications including pollutant removal, bioremediation, and valuable biomolecule production. However, despite the promising genomic features, further experimental validation and optimization are necessary for sustainable wastewater treatment.

## Data Availability

The complete genomic data of *Rhodopseudomonas* sp. P1 were deposited in the NCBI database under accession number CP088006.

## Supplementary Materials

The following supporting information can be downloaded at: https://www.mdpi.com/article/10.3390/life15050754/s1, Figure S1: Subsystem categories and feature distribution of P1 genome; Figure S2: A neighbor-joining phylogenetic tree based on 16S rRNA gene sequences of strain P1 compared to the type strains of *Rhodopseudomonas* species; Figure S3: A maximum parsimony phylogenetic tree based on 16S rRNA gene sequences of strain P1 compared to type strains of *Rhodopseudomonas* species; Table S1: Genome annotation of P1 genome; Table S2: Genomic Islands found in P1 genome using Island viewer 4; Table S3: List of CARD resistance gene identifiers of P1 genome; Table S4: Summary of prophage regions identified in P1 genome; Table S5: CAZy annotation of P1 genome; Table S6: Predicted biosynthetic gene clusters (BGCs) of P1 genome; Table S7: HMMs of MicroTrait Bioelement families of P1 genome.
